# Efficacy of stylet angulation at the holding position during tracheal intubation with a videolaryngoscope: a randomized controlled trial

**DOI:** 10.1038/s41598-021-00115-x

**Published:** 2021-10-20

**Authors:** Ryo Wakabayashi, Yuki Shiko, Tomofumi Kodaira, Yuko Shiroshita, Hitomi Otsuka, Kosuke Baba, Norimasa Hishinuma

**Affiliations:** 1 Department of Anesthesia, Hokushin General Hospital, 1-5-63, Nishi, Nakano, Nagano, 383-8505 Japan; 2 Department of Integrated Science and Engineering for Sustainable Society, Chuo University, Tokyo, Japan

**Keywords:** Health care, Medical research

## Abstract

The aim of this randomized controlled trial was to determine the efficacy of stylet angulation at the holding position during tracheal intubation with a McGRATH MAC videolaryngoscope. Patients were randomized to a group for intubation without stylet angulation at the holding position (non-angulation group) and to a group for intubation with stylet angulation at the holding position (angulation group). The primary outcome was the time for placement of the tracheal tube. Sixty patients were analyzed. The mean (standard deviation) times for tube placement were 21.3 (5.6) s in the non-angulation group and 16.9 (3.8) s in the angulation group (*P* < 0.001). The scores of operator's perception of difficulty in tube delivery, number of attempts for tube delivery, and degrees of extension, abduction, internal rotation of the right upper arm and extension of the right wrist during tube placement in the angulation group were significantly smaller than those in the non-angulation group (*P* < 0.001, *P* = 0.002, *P* < 0.001, *P* < 0.001, *P* < 0.001, *P* < 0.001, respectively). Our results suggest that stylet angulation at the holding position improves maneuverability of the tracheal tube and enables easy, smooth, and swift tube placement during tracheal intubation with a McGRATH MAC videolaryngoscope.

## Introduction

Failed or difficult tracheal intubation is associated with complications including increased risk of hypertension, hypoxemia, unexpected admissions to the intensive care unit and death^1–3^. Difficulties during routine intubation in the operating room occur in 1–6% of cases and intubation failure occurs in 0.1–0.3% of cases^4–6^. Videolaryngoscopy improves the laryngeal view, reduces intubation failure, and makes intubation easier compared with conventional direct laryngoscopy^6^. Thus, videolaryngoscopes are now the first choice or default devices for some anesthetists^7^. However, the good laryngeal view provided by videolaryngoscopes does not always guarantee successful or easy intubation^8–10^.

Tracheal intubation has 3 steps: laryngeal visualization, delivery of the tracheal tube to the glottic opening, and advancement of the tube into the trachea. Although gaining a view of the glottis is the easy part when using a videolaryngoscope^11^, tube delivery to the glottis is often difficult with a videolaryngoscope because oral, pharyngeal, and laryngeal axes are not aligned and thus the tip of the tube must pass around an acute angle to enter the larynx^12–14^. An intubating stylet is widely used to assist manipulation of the tube tip^12,13^ because maneuverability of the tube tip is necessary to allow it to advance beyond the glottic opening into the trachea^15^. The optimal shape of the distal segment of a stylet in tracheal intubation with a videolaryngoscope has been investigated in previous studies^14,16,17^. In contrast, the efficacy of stylet angulation of the proximal segment has not been documented in detail^18,19^.

**Figure 1:**
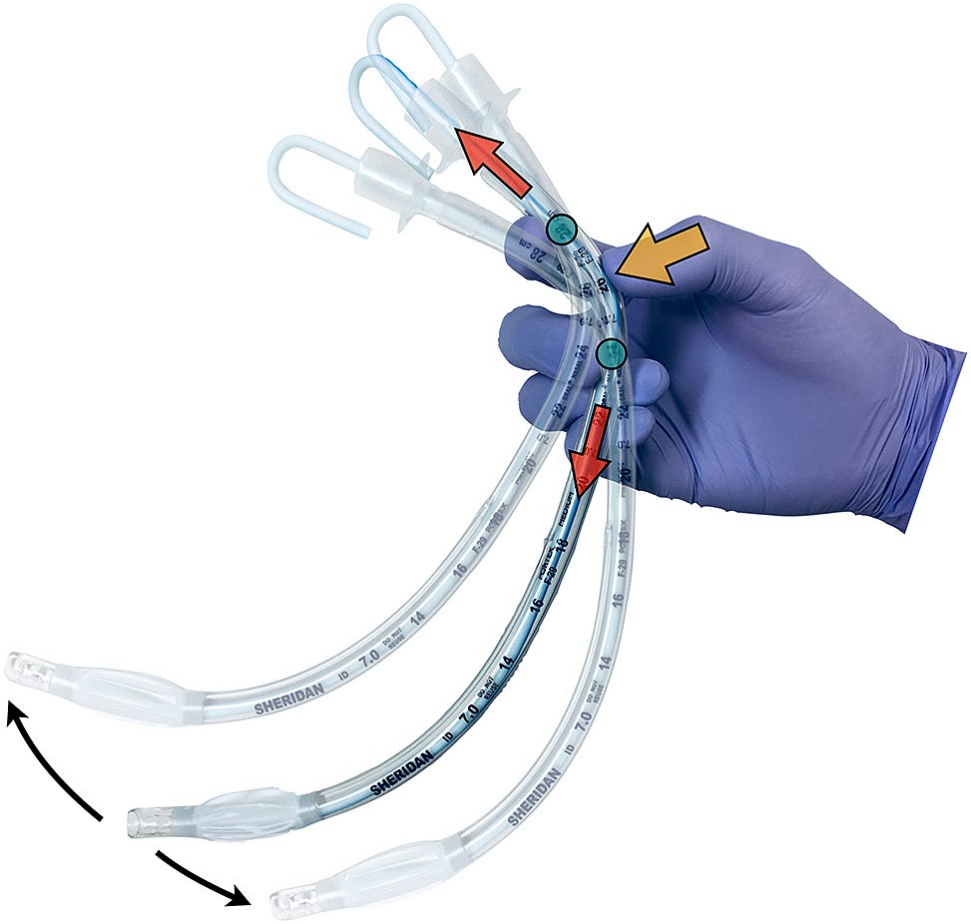
Effect of stylet angulation in the sagittal plane at the operator's holding position. The holding force of the right thumb (yellow arrow) is divided into vectors in a longitudinal direction (red arrows) and acts on the tip of the tracheal tube by using the index or middle finger as a fulcrum (green circles) during the entire process of placement of the tracheal tube. The tube tip can be efficiently and precisely operated by finger movement that is suitable for fine manipulation, and backward levering movement of the right arm, which is usually required for operation of the tube tip to enter the larynx, may be reduced.

When angulating a stylet in the sagittal plane at the operator’s holding position, the holding force of the right thumb is divided into vectors in a longitudinal direction and acts on the tip of the tracheal tube by using the index or middle finger as a fulcrum during the entire process of placement of the tracheal tube (Fig. 1). Therefore, the tube tip can be efficiently and precisely operated by finger movement that is suitable for fine manipulation^20,21^, and backward levering movement of the right arm, which is usually required for operation of the tube tip to enter the larynx^22^, may be reduced. We hypothesized that stylet angulation at the holding position would improve maneuverability of the tracheal tube and contribute to easy, smooth, and swift tube placement during intubation using a videolaryngoscope. The aim of this study was to determine the efficacy of stylet angulation at the holding position in tracheal intubation with a McGRATH MAC videolaryngoscope (Medtronic, Dublin, Ireland).

## Methods

### Study design and patient enrollment

All methods were carried out in accordance with relevant guidelines and regulations. This prospective, randomized, single-blinded, parallel groups study was approved by the Institutional Review Board of the Hokushin General Hospital (approval number: 2017016) and then the protocol was registered at the University Hospital Medical Information Network in Japan (identification number: UMIN000030204; date of registration: 30/11/2017). After obtaining written informed consent at the time of preoperative anesthetic evaluation, consecutive patients aged 20 years or older with American Society of Anesthesiologists (ASA) physical status classification 1 or 2 who were scheduled to undergo elective surgery under general anesthesia and who required oral tracheal intubation were enrolled. Exclusion criteria included the need for rapid sequence induction, known difficult mask ventilation or tracheal intubation, cervical spine instability or cervical myelopathy, symptomatic asthma or reactive airway disease, uncontrolled hypertension, an aneurysm, a history of ischemic heart disease, gastric reflux, stroke or cerebral hemorrhage, and inability to give consent. Eligible patients were randomly allocated to a group for intubation using a malleable stylet without stylet angulation at the holding position (non-angulation group) and to a group for intubation using a malleable stylet with stylet angulation at the holding position (angulation group) in a 1:1 ratio using computer-generated random numbers with a fixed block size of 4. Randomization was stratified according to sex, body mass index (BMI) and airway difficulty score. The allocation was sealed in an opaque envelope, and a study assistant opened the sealed envelope before induction of anesthesia and provided the designated stylet formation according to the group allocation. Because of difficulties with blinding of the stylet formation, intubation operators and study assistants could not be blinded.

### Preoperative evaluation and anesthetic technique

During the preoperative anesthetic evaluation, patient characteristics including age, sex, ASA physical status, height, weight, and BMI were recorded. Airway characteristics including thyromental distance, Mallampati score, mouth opening, neck mobility and upper incisors were also evaluated, and the airway difficulty score^23^ as an indicator of difficulty in airway management was calculated.

In the operating room, patients were monitored by an electrocardiogram, noninvasive blood pressure measurement, pulse oximetry, and capnography. An incompressible headrest with a height of 7 cm was placed beneath the patient’s head to obtain a sniffing position. The entire bed remained horizontal and the patient nose–floor distance was set at 100 cm. All of the patients received preoxygenation with an anesthetic circuit delivering oxygen at a flow of 6 L/min to achieve fractional end-tidal oxygen of at least 0.9. General anesthesia was induced with remifentanil (0.1–0.2 μg/kg/min) and propofol (1.5–2.0 mg/kg). After obtaining loss of consciousness, the patient's lungs were ventilated with a bag and mask, and rocuronium (0.6 mg/kg) was administered to facilitate tracheal intubation. The neuromuscular blockade was monitored using train-of-four stimulation of the ulnar nerve (TOF Watch; Organon, Dublin, Ireland), and a loss of train-of-four response was confirmed before laryngoscopy.

### Tracheal intubation

**Figure 2:**
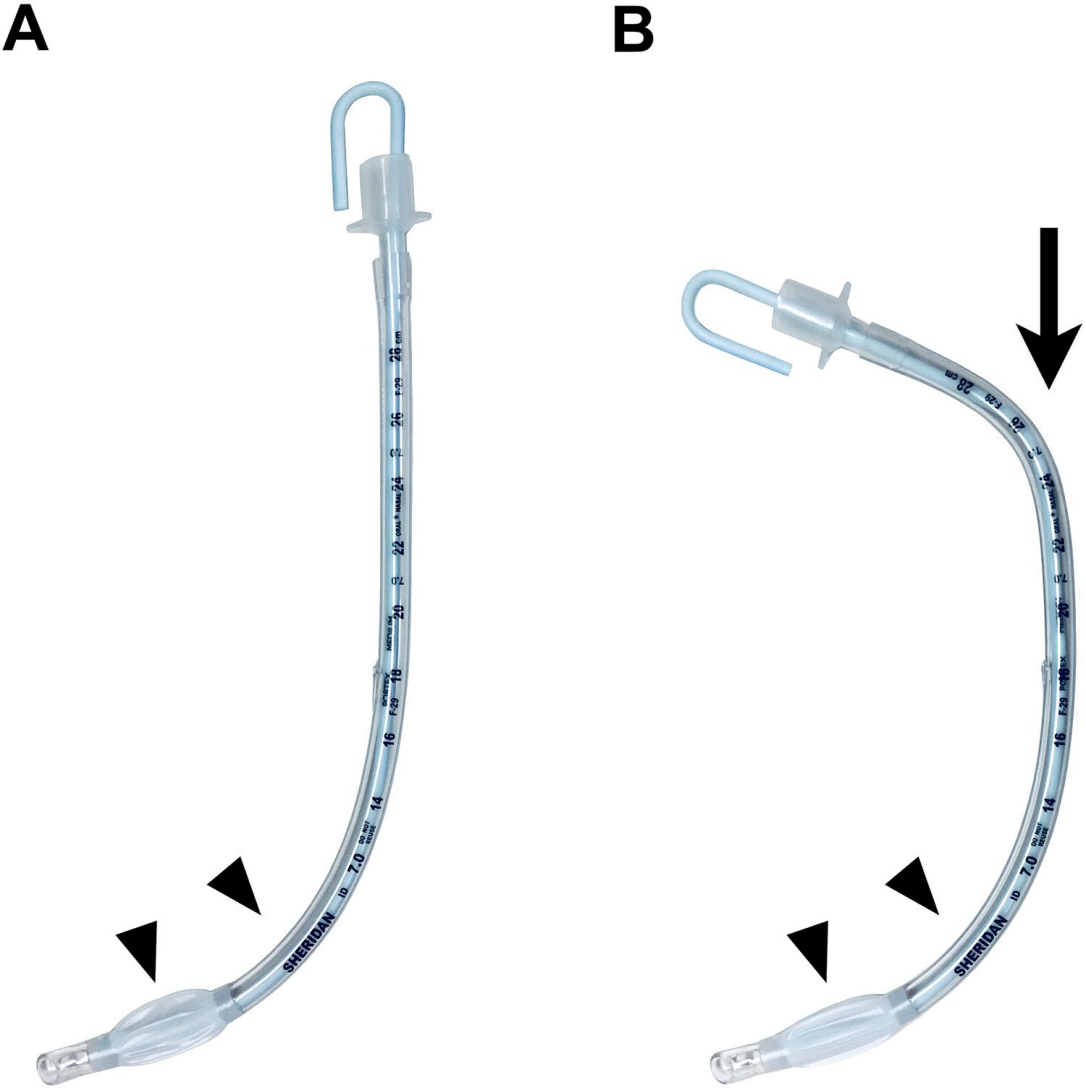
Stylet formation in each group in which tracheal intubation was performed using a malleable stylet with or without angulation at the operator's holding position. (**A**) In the group without stylet angulation at the holding position, the stylet was only curved from the distal tip of the tracheal tube to 15.0 cm in alignment with the curvature of a McGRATH MAC disposable laryngoscope blade (arrowheads). (**B**) In the group with stylet angulation at the holding position, the distal segment of the stylet was curved as in the group without stylet angulation at the holding position (arrowheads) and 8.0 cm below the proximal tip was secondarily angulated 60° in the sagittal plane, which was configured to be the operator's holding position (arrow).

A McGRATH MAC videolaryngoscope was used for all patients. McGRATH MAC 3 and 4 disposable videolaryngoscope blades (Medtronic, Dublin, Ireland) were used for women and for men, respectively. Tracheal tubes (SHERIDAN/CF; Teleflex, Wayne, PA, USA) with an internal diameter of 7.0 mm for women and with an internal diameter of 8.0 mm for men were used to intubate the trachea. Malleable stylets (PORTEX intubation stylet; Smiths Medical, Minneapolis, MN, USA) with an outer diameter of 4.0 mm were used in all patients. Standardized assistants who were blinded to the purpose of this study altered the stylet to a specified shape, prepared two types of stylet-tracheal tubes as shown in Fig. 2 and applied a water-based lubricant before tracheal intubation. For both groups, the tracheal tubes were held at 8.0 cm below the proximal tip with the right thumb, index finger, and middle finger in the same way. Tracheal intubation was conducted in order by one of three anesthetists who were skilled at intubation using a McGRATH MAC videolaryngoscope. Prior to the study, operators A (non-expert), B (expert), and C (expert) experienced tracheal intubation with stylet angulation at the holding position for 20, 10, and 10 times, respectively. Videolaryngoscopy was performed with a McGRATH MAC videolaryngoscope and the blade tip was placed in the vallecula. When an optimal view of the glottis had been displayed, the Cormack–Lehane grade and percentage of glottic opening (POGO) score^24^ were recorded and then the tracheal tube was delivered to the glottis. Patients with a Cormack–Lehane grade 3 or 4 were excluded in terms of safety. Unsuccessful intubation was defined as an excess of time from insertion of the distal tip of the videolaryngoscope blade into the oral cavity to the appearance of partial pressure of end-tidal exhaled carbon dioxide trace over 120 s or a reduction of oxygen saturation below 95%. The intervention was terminated after two unsuccessful intubations and then an alternative device was used to intubate the trachea. The operator’s motion was recorded by two cameras. According to the method of a previous study^25^, camera 1 recorded the side view of the operator. Camera 2 was positioned at a 90° angle to camera 1 to record the front view of the operator.

### Outcomes

The primary outcome was the time for placement of the tracheal tube, defined as the time from passage of the distal tip of the tracheal tube past the incisors to the appearance of an end-tidal carbon dioxide trace. Secondary outcomes included first-pass success rate for tracheal intubation, operator's perception of difficulty in delivery of the tracheal tube expressed using a 11-point ordinal score with 0 representing easiest and 10 representing most difficult, intubation difficulty scale (IDS) score^26^, number of attempts for delivery of the tracheal tube, and deflections of the right upper arm, lower arm, and wrist during placement of the tracheal tube. Number of delivering attempts was added if withdrawal and re-advancement of the tracheal tube in the cavity of pharynx occurred. Deflections of the right upper arm, lower arm, and wrist during the period from passage of the distal tip of the tracheal tube past the incisors to achievement of delivery of the tube tip to the glottis were measured by using video data recording the operator’s motion. The outcomes were evaluated by two assistants who were not involved in the study design.

### Statistical analysis

Categorical data are presented as numbers (proportion), and continuous data are presented as means (standard deviation) if normally distributed and otherwise as medians (interquartile range). Normality of the continuous variables was checked by using the Shapiro–Wilk *W*-test. Equality of variances was assessed by using Levene’s test.

The primary outcome was the time for placement of the tracheal tube. We compared the time for placement of the tracheal tube between the non-angulation and angulation groups using Welch's t-test. In a secondary analysis, the differences in first-pass success rates for tracheal intubation between the two groups were evaluated using the chi-squared test. The differences in the operator's perception of difficulty in delivery of the tracheal tube, IDS score, and number of attempts for delivery of the tracheal tube between the groups were compared using the Mann–Whitney *U*-test. The differences in deflections of the right upper arm, lower arm, and wrist during placement of the tracheal tube were evaluated using Student’s t-test, Welch's t-test, or the Mann–Whitney *U*-test.

All statistical tests were 2-tailed, and a *P* value of less than 0.05 was considered statistically significant for the primary and secondary outcomes with no adjustment applied. All statistical analyses were performed using GraphPad Prism 7 (GraphPad Software, La Jolla, CA, USA).

### Sample size estimation

The sample size was determined by using our preliminary data. In our preliminary study, the mean (standard deviation) time for placement of the tracheal tube was 25 (10) s when using a stylet without angulation at the holding position. Assuming that stylet angulation at the holding position would reduce the time for tube placement for 10 s, with type I error set at 5% and type II error set at 5%, 28 patients were required in each group (ie, 56 patients in total). We decided to recruit 30 patients for each group to compensate for dropouts and missing data. We thus scheduled assessment for eligibility of about 80 patients to accommodate a 25% exclusion rate.

## Results

**Figure 3:**
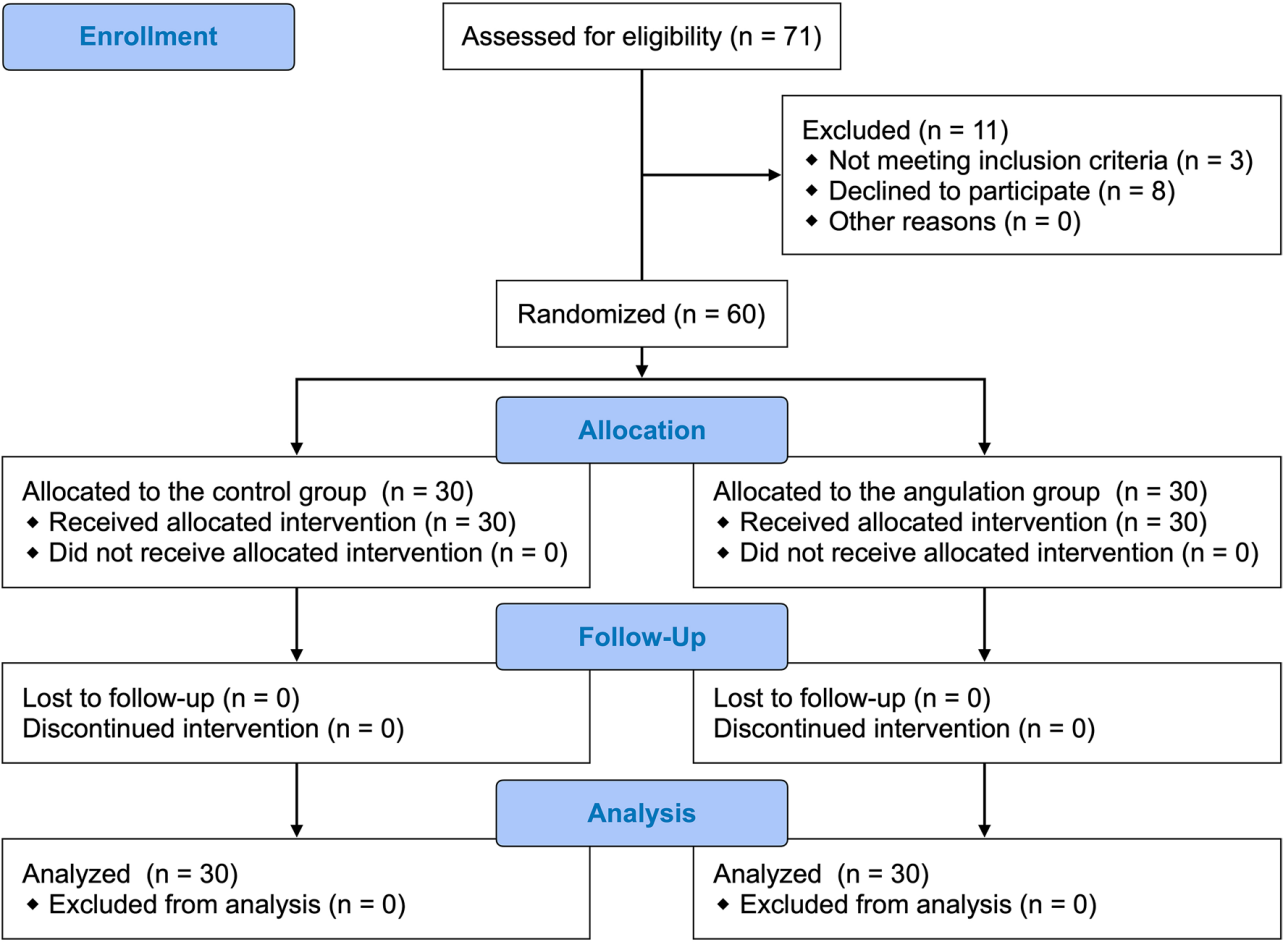
CONSORT study flow diagram.

Sixty patients were included in this study and were randomly allocated to each group between December 2017 and March 2018 (Fig. 3). Baseline and airway characteristics in the two groups are shown in Table 1. The primary and secondary outcomes are summarized in Table 2. The times for placement of the tracheal tube in both groups were normally distributed. The mean (standard deviation) times for placement of the tracheal tube were 21.3 (5.6) s in the non-angulation group and 16.9 (3.8) s in the angulation group (*P* < 0.001). The median (interquartile range) scores of the operators’ perception of difficulty in tube delivery were 3 (3–4) in the non-angulation group and 2 (1–3) in the angulation group (*P* < 0.001). The median (interquartile range) numbers of attempts for delivery of the tracheal tube were 2 (1–3) in the non-angulation group and 1 (1–1) in the angulation group (*P* = 0.002). The mean (standard deviation) degrees of right upper arm extension during placement of the tracheal tube were 31 (8) in the non-angulation group and 15 (5) in the angulation group (*P* < 0.001). The median (interquartile range) degrees of right upper arm abduction were 18 (13–24) in the non-angulation group and 9 (7–11) in the angulation group (*P* < 0.001). The median (interquartile range) degrees of right upper arm internal rotation were 38 (26–43) in the non-angulation group and 18 (13–21) in the angulation group (*P* < 0.001). The median (interquartile range) degrees of right wrist extension were 31 (25–35) in the non-angulation group and 14 (10–18) in the angulation group (*P* < 0.001). There was no difference in the success rate of first-pass intubation or IDS score between the two groups. Right lower arm flexion and pronation and right wrist radial deviation during placement of the tracheal tube did not differ between the two groups.

**Table 1:** Baseline and airway characteristics of study patients. Data are expressed as mean (standard deviation), number (proportion), or median (interquartile range). *ASA* American Society of Anesthesiologists.

	Non-angulation (*n* = 30)	Angulation (*n* = 30)
Age (years)	61 (18)	61 (15)
Male sex	14 (47%)	16 (53%)
ASA physical status		
1	12 (40%)	11 (37%)
2	18 (60%)	19 (63%)
Height (cm)	161 (10)	161 (9)
Weight (kg)	60 (12)	60 (12)
Body mass index (kg/m^2^)	23 (3)	23 (4)
Airway difficulty score	7 (6–7)	7 (6–8)
Cormack–Lehane grade		
1	15 (50%)	16 (53%)
2a	15 (50%)	14 (47%)
Percentage of glottic opening score	95 (70–100)	100 (70–100)
Intubation operator		
A	13 (43%)	13 (43%)
B	7 (23%)	7 (23%)
C	10 (33%)	10 (33%)

**Table 2:** Primary and secondary outcomes. Data are expressed as mean (standard deviation), number (proportion), or median (interquartile range).

	Non-angulation (*n* = 30)	Angulation (*n* = 30)	*p* value
**Primary outcome**			
Time for placement of the tracheal tube (s)	21.3 (5.6)	16.9 (3.8)	< 0.001
**Secondary outcomes**			
First-pass intubation success	30 (100%)	30 (100%)	> 0.999
Operator’s perception of difficulty in tube delivery	3 (3–4)	2 (1–3)	< 0.001
Intubation difficulty scale score	1 (0–2)	0 (0–1)	0.290
Number of attempts for tube delivery	2 (1–3)	1 (1–1)	0.002
Right upper arm deflection (°)			
Extension	31 (8)	15 (5)	< 0.001
Abduction	18 (13–24)	9 (7–11)	< 0.001
Internal rotation	38 (26–43)	18 (13–21)	< 0.001
Right lower arm deflection (°)			
Flexion	25 (8)	26 (8)	0.629
Pronation	130 (26)	129 (25)	0.821
Right wrist deflection (°)			
Extension	31 (25–35)	14 (10–18)	< 0.001
Radial deviation	7 (2)	7 (2)	0.868

## Discussion

This is the first study to elucidate the efficacy of stylet angulation at the holding position for placement of the tracheal tube in tracheal intubation using a McGRATH MAC videolaryngoscope. In this study, stylet angulation at the holding position shortened the time for tube placement, improved the operator’s perception of difficulty in delivery of the tracheal tube, reduced the number of delivering attempts, and reduced deflections of the operator’s right arm during tube placement.

We demonstrated that stylet angulation at the holding position decreased extension, abduction and internal rotation of the right upper arm and extension of the right wrist during placement of the tracheal tube, indicating an ergonomic improvement during tube placement. Additionally, the reduced number of delivering attempts of the tracheal tube indicates that stylet angulation at the holding position enables precise delivery of the tracheal tube towards the glottic opening. Taken together, the results suggest that stylet angulation at the holding position improves maneuverability of the tracheal tube during tracheal intubation with a McGRATH MAC videolaryngoscope.

The shortened time for tube placement and improved operator’s perception of difficulty in tube delivery suggest that stylet angulation at the holding position eases placement of the tracheal tube. The time for placement of the tracheal tube was chosen as the primary outcome because we predicted that easier tube placement would shorten the time for tube placement. We assumed that stylet angulation at the holding position would reduce the time for tube placement for 10 s as a clinically meaningful difference^27^. However, the time for placement of the tracheal tube was shortened by only 4.4 s and the difference was clinically irrelevant. Less experience of stylet angulation at the holding position by the operators may have led to this result^28^.

The success rate of first-pass intubation and IDS score did not differ between the two groups. These results might be because tracheal intubation in the non-angulation group was already easy when using a McGRATH MAC videolaryngoscope in the operating room. Indeed, the success rate of first-pass intubation or IDS score in the non-angulation group was consistent with previous studies using a McGRATH MAC videolaryngoscope in the operating room in which the success rate was 97.5–100%^29,30^ or IDS score was 0–1^29,31^. If we had investigated in difficult airway settings, there might have been a larger effect on the difficulty in tracheal intubation. Further studies are required to determine the effect of stylet angulation at the holding position in difficult airway settings such as tracheal intubation in an intensive care unit or emergency department^32^.

Our study has some limitations. First, we could not blind the intubation operators to the stylet form being used, and Hawthorne effects might thus have affected the performance during tracheal intubation^33^. Second, we did not enroll patients who had an anticipated difficulty airway. Thus, the results of our study may not be applicable to patients with a difficult airway condition. Finally, only a McGRATH MAC videolaryngoscope was used in this study. Whether stylet angulation at the holding position is useful when using a direct laryngoscope or other laryngoscope models is unknown.

In conclusion, our results suggests that stylet angulation at the holding position improves maneuverability of the tracheal tube and enables easy, smooth, and swift tube placement in tracheal intubation with a McGRATH MAC videolaryngoscope.

## Data Availability

The datasets generated during and/or analyzed during the current study are not publicly available due to concerns about backtracking of personal information of study subjects but are available from the corresponding author on reasonable request.
